# PPM1G regulates hepatic ischemia/reperfusion injury through STING‐mediated inflammatory pathways in macrophages

**DOI:** 10.1002/iid3.1189

**Published:** 2024-02-19

**Authors:** Dadi Peng, Zuotian Huang, Hang Yang, Yunhai Luo, Zhongjun Wu

**Affiliations:** ^1^ Department of Hepatobiliary Surgery The First Affiliated Hospital of Chongqing Medical University Chongqing China; ^2^ Department of Hepatobiliary Pancreatic Tumor Center Chongqing University Cancer Hospital Chongqing China

**Keywords:** cell activation, inflammatory pathways, ischemia/reperfusion injury, macrophage polarization, monocytes/macrophages, protein kinases/phosphatases

## Abstract

**Background:**

Ischemia/reperfusion injury (IRI) is generally unavoidable following liver transplantation. Here, we investigated the role of protein phosphatase, Mg^2+^/Mn^2+^ dependent 1G (PPM1G) in hepatic IRI.

**Methods:**

Hepatic IRI was mimicked by employing a hypoxia/reperfusion (H/R) model in RAW 264.7 cells and a 70% warm ischemia model in C57BL/6 mice, respectively. In vitro, expression changes of tumor necrosis factor‐α and interleukin were detected by quantitative real‐time polymerase chain reaction (qRT‐PCR), western blot analysis, and enzyme‐linked immunosorbent assay. The protein expressions of PPM1G and the stimulator of interferon genes (STING) pathway components were analyzed by western blot. Interaction between PPM1G and STING was verified by coimmunoprecipitation (CO‐IP). Immunofluorescence was applied for detection of p‐IRF3. Flow cytometry, qRT‐PCR and western blot were utilized to analyze markers of macrophage polarization. In vivo, histological analyses of mice liver were carried out by TUNEL and H&E staining. Changes in serum aminotransferases were also detected.

**Results:**

Following H/R intervention, a steady decline in PPM1G along with an increase in inflammatory cytokines in vitro was observed. Addition of plasmid with PPM1G sequence limited the release of inflammatory cytokines and downregulated phosphorylation of STING. CO‐IP validated the interaction between PPM1G and STING. Furthermore, inhibition of PPM1G with lentivirus enhanced phosphorylation of STING and its downstream components; meanwhile, p65, p38, and Jnk were also surged to phosphorylation. Expression of INOS and CD86 was surged, while CD206, Arg‐1, and IL‐10 were inhibited. In vivo, PPM1G inhibition further promoted liver damage, hepatocyte apoptosis, and transaminases release. Selective inhibition of STING with C‐176 partially reversed the activation of STING pathway and inflammatory cytokines in vitro. M1 markers were also suppressed by C‐176. In vivo, C‐176 rescued liver damage and transaminase release caused by PPM1G inhibition.

**Conclusion:**

PPM1G suppresses hepatic IRI and macrophage M1 phenotype by repressing STING‐mediated inflammatory pathways.

## Graphical abstract

PPM1G was downregulated in hepatic IRI, thus promoting STING phosphorylation. PPM1G restricts macrophage M1 polarization.

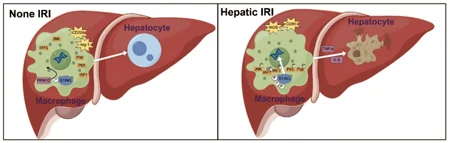

## 1 INTRODUCTION

Liver transplantation has been the optimal choice for a range of end‐stage liver diseases.^1^ Since the quality of the donor liver has a detrimental influence on the prognosis of the recipient, it is crucial to preserve its physiological state during surgery. However, ischemia/reperfusion injury (IRI), a prevalent pathogenic event in liver transplantation that provokes a complex inflammatory response and compromises liver function, is currently unavoidable.^2^ Hepatic IRI is essentially triggered by a wide range of molecular mechanisms, including oxidative stress, mitochondrial dysfunction, the inflammatory response, and immune surveillance; however, effective preventive and therapeutic methods are still lacking.^3^, ^4^, ^5^ As a result, comprehending the underlying molecular mechanisms of hepatic IRI is imperative, although we know virtually little about these mechanisms.

Kupffer cells (KCs), specialized macrophages that reside in hepatic sinusoids, undergo different phenotypic transformations in different immune microenvironments,^6^, ^7^ with the M1 phenotype acting as an exacerbator of inflammation, whereas the M2 phenotype acts as a tissue repairer and reliever of inflammation. KCs dominate M1 macrophages in hepatic IRI, and any effective approaches reversing KCs from M1 polarization to M2 polarization should ameliorate ischemic hepatic impairment.^8^, ^9^, ^10^ Therefore, in‐depth investigations of the fundamental processes that modify KC polarization are worth exploring.

Protein phosphatase Mg^2+^/Mn^2+^ dependent 1G (PPM1G) is one of several members of the PPM family that functions as a Mg^2+^ or Mn^2+^ bonded single‐subunit enzyme. Previously, the majority of research on PPM1G has focused on its participation in cell cycle regulation, inflammatory reactions, and tumor progression, during which PPM1G spontaneously dephosphorylates target proteins or bind proteins and nucleic acids through its negatively charged unique long loop/linker L4.^11^, ^12^, ^13^ For instance, PPM1G blocks the initiation of protein translation by dephosphorylating 4E‐BP1 and p27 at T198, resulting in decreased cell volume and protein density.^14^, ^15^ It also degrades overexpressed HIF‐1 via the proteasomal pathway under acute oxidative stress.^16^ Recently, the significance of PPM1G in liver diseases has been elucidated, as PPM1G complexes with WWP2 promote liver fibrosis by overactivating the Notch3/HES1 pathway and contributing to the growth of hepatocellular carcinoma by modulating SRSF3 phosphorylation.^17^, ^18^, ^19^ However, its importance in hepatic IRI still needs to be clarified.

In the present work, we explored the specific impact of PPM1G on hepatic IRI through STING pathway via an approach involving the dephosphorylation of STING and the participation of PPM1G in limiting M1 polarization in macrophages, thus revealing the protective role of PPM1G in hepatic IRI.

## 2 MATERIALS AND METHODS

### 2.1 Cell and H/R model

RAW 264.7 cells, a classic surrogate of KCs,^20^, ^21^ were purchased from the Cell Bank of the Type Culture Collection of the Chinese Academy of Sciences. RAW 264.7 cells were grown in Dulbecco's modified Eagle's medium (Gibco) supplemented with 10% fetal bovine serum (Gibco), 100 U/mL penicillin (Beyotime) and 0.1 mg/mL streptomycin (Beyotime). A cell incubator (Thermo) set into 5% CO_2_ at 37°C was used to incubate RAW 264.7 cells.

The hypoxia/reperfusion (H/R) model was applied as a classic in vitro hepatic IRI model.^9^ Hypoxia was preconditioned using a tri‐gas incubator (Thermo) supplemented with N_2_ (94%), O_2_ (1%), and CO_2_ (5%). To explore an appropriate H/R combination, hypoxia was applied for 1, 6, 12, or 24 h, while reoxygenation was applied for 1, 3, 6, or 12 h.

### 2.2 Mice and IRI model

C57BL/6J male mice aged 6–8 weeks and 25–28 g were obtained from the Chongqing Medical University Laboratory Animals Center. The Animal Care and Use Committee of Chongqing Medical University authorized all animal formalities. All animal experiments adhered to the National Institutes of Health guidance for the care and use of laboratory animals (NIH Publications No. 8023, amended 1978). A pathogen‐free environment with a 12‐h light/dark cycle, relative humidity between 60% and 65%, and a temperature of 23°C was used for mouse housing.

As described previously, a 70% warm liver I/R model was established.^9^ Pentobarbital sodium (45 mg/kg) was injected intraperitoneally to anesthetize the animals before performing a laparotomy. Hepatic ischemia injury was achieved by placing a clamp across the hepatic artery and portal vein. The clamp was removed after 1 h of ischemia to allow for 6 h of reperfusion. Mice in the sham group underwent the exact procedure but without any liver vascular clamping.

### 2.3 Cell transfection

Plasmids containing PPM1G (+) for overexpression or the corresponding NC sequence (Genepharma) were transfected into RAW 264.7 cells. Opti‐MEM (Gibco) and Lipofectamine 2000 reagents (Beyotime) were used according to the manufacturer's instructions. The validity of the transfection efficiency is shown in Supporting Information S1: Figure S1A.

RAW 264.7 cells were incubated with PPM1G‐specific shRNA or negative control (NC) (Genepharma) packaged in lentiviral vectors according to the manufacturer's instructions. Eventually, 6 μg/mL puromycin was chosen for stable culture of RAW 264.7 cells infected with lentivirus. The efficiency of the PPM1G shRNAs was validated (Supporting Information S1: Figure S1B). The sequence of the PPM1G shRNAs used was 5′‐GCA AGC TTC AGA AGG CTT TAC‐3′.

C‐176 (MedChemExpress) was used as a selective stimulator of interferon genes (STING) inhibitor^22^ and was incubated with RAW 264.7 cells for 6 h before H/R treatment.

### 2.4 Mouse transfection

PPM1G shRNA was injected into the tail veins of mice 14 days before IRI. The transfection efficiency was verified by quantitative real‐time polymerase chain reaction (qRT–PCR) and western blot analysis (Supporting Information S1: Figure S1C,D). A total of 750 nmol C‐176 in 200 μL of corn oil^22^ or vehicle (1% DMSO + corn oil) per mouse was administered intraperitoneally 30 min before IRI.

### 2.5 qRT‐PCR

Total RNA was extracted from RAW 264.7 cells using TRIzol (Invitrogen) and then reverse‐transcribed to cDNA using the PrimeScript™ RT Reagent Kit with gDNA Eraser (Takara). qRT‐PCR was performed using a CFX96 Real‐Time PCR Detection System (Bio‐Rad). The relative mRNA levels were quantified by TB Green Premix Ex Taq II (Takara) and adjusted to the level of β‐actin. Primer sequences were listed: PPM1G forward: 5′‐CTT GTG ACG GCA TCT GGA ATG‐3′; PPM1G reverse: 5′‐TGC ACG TCA TGT TGT‐ CAC AC‐3′; interleukin (IL)−6 forward: 5′‐CTC TGG GAA ATC GTG GAA ATG‐3′; IL‐6 reverse: 5′‐AAG TGC ATC ATC GTT GTT CAT ACA‐3′; tumor necrosis factor (TNF)‐α forward: 5′‐TAT GGC‐TCA GGG TCC AAC TC‐3′; TNF‐α reverse: 5′‐GGA AAG CCC ATT TGA GTC CT‐3′; Arg1 forward: 5′‐CTG CCT GCT TTC TGA GTG CTG AG‐3′; Arg1 reverse: 5′‐CCT GTG GTT CCG ATA AGT GCT‐ TCC‐3′; CD206 forward: 5′‐GGA ATC AAG GGC ACA GAG TTA‐3′; CD206 reverse: 5′‐ATT GTG‐ GAG CAG ATG GAA‐3′; IL‐10 forward: 5′‐AAC CCA GGC ACA TCC GAA AAG C‐3′; IL‐10 reverse: 5′‐AGA GAC TAC GCA GAG ACC ACA GAC‐3′; INOS forward: 5′‐GGT CTT TGA AAT CCC TCC‐ TGA‐3′; INOS reverse: 5′‐AGC TCC TGG AAC CAC TCG TA‐3′; CD86 forward: 5′‐AAA GTT GGT‐TCT GTA CGA GCA C‐3′; CD86 reverse: 5′‐GGC CCA GGT ACT TGG CAT T‐3′; β‐actin forward: 5′‐GGC TGT ATT CCC CTC CAT CG‐3′; β‐actin reverse: 5′‐CCA GTT GGT AAC AAT GCC ATG T‐3′. All the samples were examined in triplicate, and the results were analyzed using the 2^−ΔΔ*Ct*^ method.

### 2.6 Western blot analysis

Total protein was extracted from RAW 264.7 cells using RIPA lysis buffer (Beyotime) plus a phosphatase inhibitor cocktail (MedChemExpress). The proteins were transferred onto polyvinylidene fluoride (PVDF) membranes after being separated via sodium dodecyl sulfate‒polyacrylamide gel electrophoresis. Blocking PVDF membranes with 5% skim milk, and then incubated at 4°C overnight with the following primary antibodies: PPM1G, stimulator of interferon gene (STING), TANK‐binding kinase 1 (TBK1), interferon regulatory factor 3 (IRF3), INOS, P38, p‐P38, Arg‐1, IL‐10, Nrf2, HO‐1 (Proteintech Group), p‐STING, p‐TBK1, p‐IRF3 (CST), p‐JNK1/2/3, CD206, P65, p‐P65, TNF‐α, IL‐6, β‐actin (Beyotime), IRF7, p‐IRF7 (Bioss), JNK1/2/3 (ABclonal). The dilution ratios of the primary antibodies are shown in Supporting Information S1: Table S1. The membranes were then incubated with the appropriate secondary antibodies before being incubated with enhanced chemiluminescence reagents (Zen BioScience) for visualization. β‐Actin served as the internal control.

### 2.7 Coimmunoprecipitation (CO‐IP) analysis

The RAW 264.7 cells, which had reached 80%–90% confluence, were disrupted using Buffer A. Buffer A was composed of 25 mM Tris–HCl, 10% glycerol, 150 mM NaCl, and 1% Triton X‐100, with a pH of 7.6. Following incubation with an anti‐PPM1G antibody or normal immunoglobulin G (IgG) (Beyotime) at 4°C overnight, the precleared protein extracts (1000 μg) were then incubated with protein A + G magnetic beads (MedChemExpress) at 4°C for 4 h. Afterward, the obtained protein complexes were washed, eluted, denatured, and subjected to western blot analysis.

### 2.8 Immunofluorescence

RAW 264.7 cells were grown on glass coverslips. After H/R treatment or transfection, the cells were fixed for 10 min with 4% paraformaldehyde. For a 1‐h blockade, goat serum was used. For 30 min of cellular permeabilization, 0.3% Triton X‐100 dissolved in goat serum was utilized. After overnight incubation with F4/80 (Abcam) and anti‐p‐IRF3 (Bioss) at 4°C, the cover slips were gently washed three times with Tris‐buffered saline with Tween (TBST) and then reincubated at 37°C for 1 h with goat anti‐rabbit (FITC‐labeled; Beyotime) or goat anti‐mouse (Cy3‐labeled; Beyotime) IgG. The nucleus was stained with 4′,6‐diamidino‐2‐phenylindole for 8 min.

### 2.9 Cytokine and aminotransferase detection

The release of TNF‐α and IL‐6 in the cell supernatant or mouse serum was detected using enzyme‐linked immunosorbent assay (ELISA) kits (Neobioscience) following the manufacturer's instructions. After the manufacturer's instructions, mouse serum alanine transaminase (ALT) and aspartate transaminase (AST) concentrations were detected by liver enzyme kits (JianCheng Bioengineering Institute). A microplate reader (Biotek) was used to quantify the absorbance of all the samples.

### 2.10 Flow cytometry analysis

Raw 264.7 cells were stained with anti‐F4/80‐FITC (BioLegend) and anti‐CD86‐PE (BioLegend) to assess the ratio of M1 macrophages to total macrophages. After being suspended as single cells in phosphate‐buffered saline (PBS), the Raw 264.7 cells were treated with TruStain FcX (anti‐mouse CD16/32) antibody (BioLegend) for Fc receptor blockade. RAW 264.7 cells were then incubated with the antibodies for 30  min in a dark room, followed by washing twice with PBS. The staining was assessed by a CytoFLEX Flow Cytometer (Beckman Coulter) and analyzed by CytExpert 2.4 software.

### 2.11 Histological analyses

Paraformaldehyde‐embedded liver slices from the mice were cut and subsequently stained. Suzuki's score was used to assess hepatic IRI pathological impairment based on the results of hematoxylin and eosin (H&E) staining. Following the manufacturer's instructions, a TUNEL kit (Beyotime) was used to measure the level of hepatic apoptosis. For observation at various magnifications, a microscope (Olympus) and ZEN2012 were used.

### 2.12 Statistical analysis

All the data are presented as the mean ± SD. The Brown–Forsythe test was performed to test for equal variances, and normally distributed data were tested with the Shapiro‒Wilk test. For parametric data, one‐way analysis of variance (ANOVA) with Tukey's post hoc test was used to evaluate significant differences among groups, while the Student *t* test was used to compare two groups. For nonparametric data, the Kruskal–Wallis test was applied. Significance was defined as a *p* value of less than .05. The results were analyzed with GraphPad Prism (version 9.3).

## 3 RESULTS

### 3.1 In vitro, the expression of PPM1G was downregulated, while that of inflammatory markers was upregulated in a hepatic H/R model

**Figure 1:**
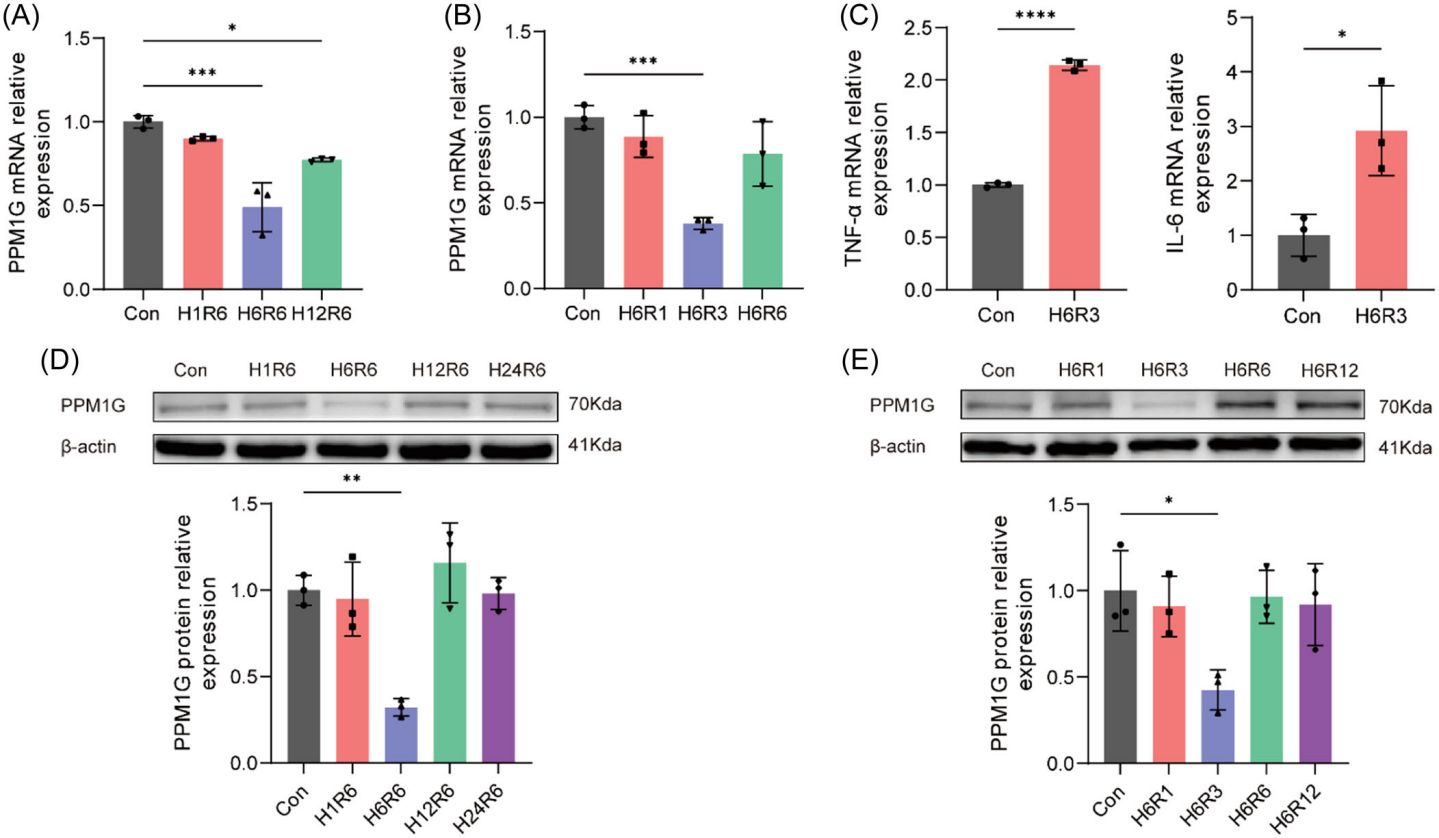
Expression of PPM1G was downregulated while inflammation markers were upregulated in hepatic hypoxia/reperfusion (H/R) in vitro model. (A) The messenger RNA (mRNA) expression of PPM1G in RAW 264.7 cells after hypoxia for 1, 6, or 12 h followed by reoxygenation for 6 h. (B) The mRNA expression of PPM1G in RAW 264.7 cells after hypoxia for 6 h and reoxygenation for 1, 3, or 6 h. (C) The mRNA expression of TNF‐α and IL‐6 in RAW 264.7 cells after hypoxia for 6 h followed by reoxygenation for 3 h. (D) The protein expression of PPM1G in RAW 264.7 cells after hypoxia for 1, 6, 12, or 24 h followed by reoxygenation for 6 h. (E) The protein expression of PPM1G in RAW 264.7 cells after hypoxia for 6 h and reoxygenation for 1, 3, 6, or 12 h. Results are expressed as the relative mean and SD ratio of three independent sets of experiments. Statistical significance was assessed by unpaired *t* test (C) and one‐way analysis of variance (ANOVA) (A, B, D, E). *p < .05, **p < .01, ***p < .001 and ****p < .0001. Con, control; H, hypoxia; R, reoxygenation.

As a classic in vitro model of hepatic IRI, the H/R model was established to examine changes in the expression of PPM1G. RAW 264.7 cells were cultured in a tri‐gas incubator to simulate a hypoxic environment, followed by reperfusion challenge. To determine the ideal time point for observing changes in PPM1G expression, we rigorously established a hypoxia and reperfusion time gradient as reported previously.^9^, ^23^ As a result, both the protein and mRNA expression of PPM1G decreased over time, reaching a trough at 6 h after hypoxia and 3 h after reoxygenation, respectively (Figure 1A,B,D,E).

Thereafter, a fixed time point of 6 h of hypoxia followed by 3 h of reoxygenation was selected as the standard for in vitro H/R intervention in subsequent experiments. Furthermore, the mRNA levels of both TNF‐α and IL‐6 were considerably greater after H/R treatment than in the control group, revealing the enhanced potency of inflammatory activity (Figure 1C).

### 3.2 Overexpression of PPM1G inhibited STING pathway activation and inflammatory cytokine release

**Figure 2:**
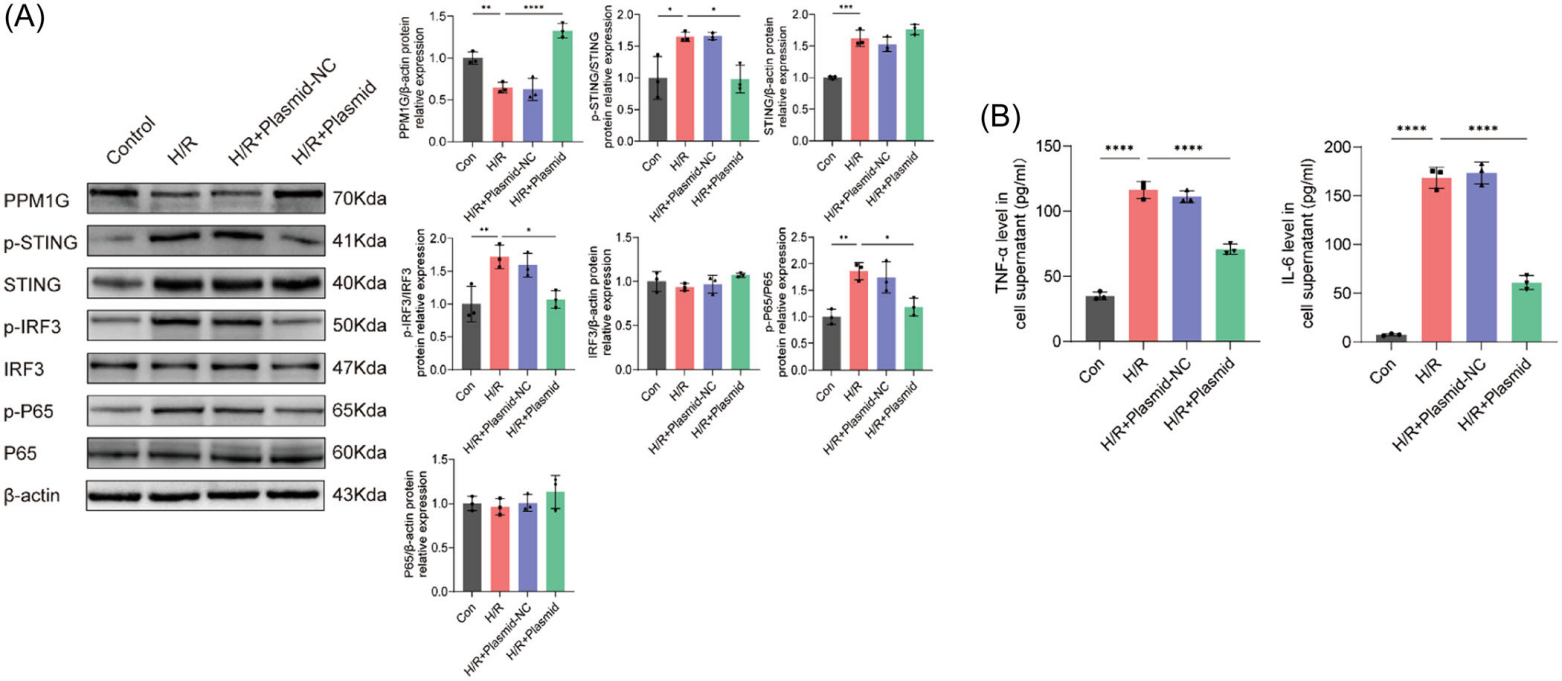
Addition of PPM1G suppressed STING pathway. (A) Immunoblotting images and quantitative analysis of PPM1G, p‐STING, STING, p‐IRF3, IRF3, p‐P65, and P65 in RAW 264.7 cells. (B) Levels of TNF‐α and IL‐6 in cell supernatant. Results are expressed as the relative mean and SD ratio of three independent sets of experiments. Statistical significance was assessed by one‐way analysis of variance (ANOVA). *p < .05, **p < .01, ***p < .001 and ****p < .0001. Con, control; H, hypoxia; NC, negative control; R, reoxygenation.

To determine the involvement of PPM1G in hepatic H/R in vitro, PPM1G was overexpressed by transfecting cells with plasmids containing PPM1G or NC sequences. After PPM1G was overexpressed, the level of inflammatory cytokines further decreased compared to that in the H/R group, while no similar effect was observed in the group treated with the NC‐sequence plasmid (Figure 2B). These findings prompted us to investigate the mechanism by which PPM1G achieves this effect. As reported recently, As reported recently, PPM1G maintains balanced STING activity, a transmembrane protein that predominantly governs cytosolic nucleic sensing and ultimately initiates both immunological and inflammatory responses downstream, by dephosphorylating at Ser366 in antivirus reaction.^24^ Therefore, we examined the protein expression of STING and several downstream components to evaluate whether hepatic IRI is affected by this particular mechanism. In accordance with our expectations, the expression of both the STING total protein and its Ser366 phosphorylated form was enhanced during H/R stimulation, while its phosphorylated form was reversely downregulated following the addition of a plasmid containing the PPM1G sequence. However, the total protein expression of STING was not affected by plasmid transfection. In addition, dephosphorylation of interferon regulatory factor 3 (IRF3), an adapter that activates STING, was promoted after PPM1G upregulation (Figure 2A). Notably, the protein expression of phosphorylated p65 followed a similar pattern as that of IRF3 (Figure 2A), implying that NF‐κB, the predominant inflammatory signaling pathway, was also stimulated in H/R. Overall, PPM1G may affect hepatic IRI via the STING pathway, which we explored in more depth in subsequent experiments.

### 3.3 Inhibition of PPM1G overactivated the STING pathway and further induced M1 macrophage polarization

**Figure 3:**
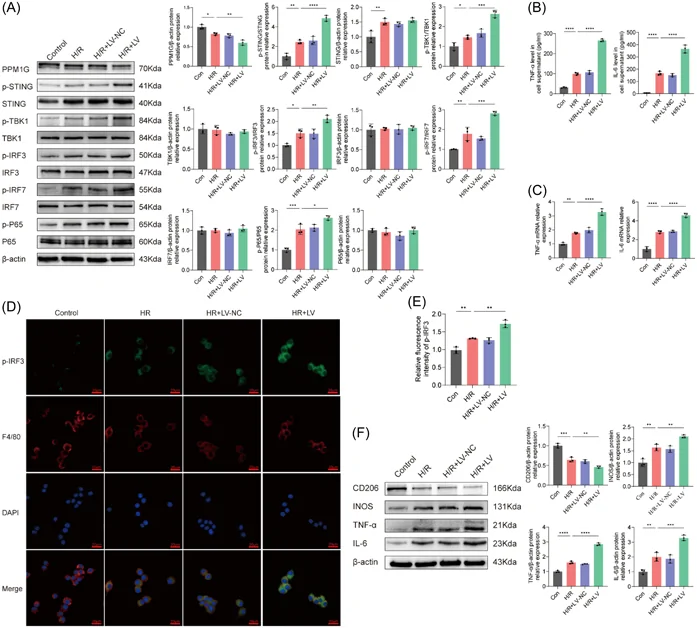
Inhibition of PPM1G overactivated STING pathway and further induced macrophage M1 polarization. (A) Immunoblotting images and quantitative analysis of PPM1G, p‐STING, STING, p‐TBK1, TBK1, p‐IRF3, IRF3, p‐IRF7, IRF7, p‐P65 and P65 in RAW 264.7 cells. (B) Levels of TNF‐α and IL‐6 in cell supernatant. (C) The mRNA expressions of TNF‐α and IL‐6 in RAW 264.7 cells. (D, E) Immunofluorescence of p‐IRF3 and F4/80 in RAW 264.7 cells. Magnification, ×200; scale bar = 20 μm. (F) Immunoblotting images and quantitative analysis of macrophage polarization markers and inflammatory factors including CD206, INOS, TNF‐α, and IL‐6 in RAW 264.7 cells. Results are expressed as the relative mean and SD ratio of three independent sets of experiments. Statistical significance was assessed by one‐way analysis of variance (ANOVA). *p < .05, **p < .01, ***p < .001 and ****p < .0001. Con, control; H, hypoxia; LV, lentivirus; mRNA, messenger RNA; NC, negative control; R, reoxygenation.

To investigate the influence of PPM1G blockade on the STING pathway and hepatic IRI, lentiviruses packaged with specific shRNAs or NC sequences were fully utilized in vitro. After PPM1G suppression, the mRNA expression, protein expression in cells and supernatant concentration of inflammatory cytokines increased further compared to those in the H/R group. However, the H/R group treated with NC‐packaged lentivirus did not exhibit the same pattern (Figure 3B,C,F). In addition, the protein expression of phosphorylated STING was more prevalent following PPM1G blockade, according to the western blot results (Figure 3A). Notably, the phosphorylation of TBK1, another adapter involved in STING activation, and IRF7, another downstream component,^25^ increased after PPM1G knockdown, which was consistent with the changes in IRF3 and P65 expression (Figure 3A). Moreover, the changing pattern in p‐IRF3 expression detected by immunofluorescence was consistent with the western blot analysis (Figure 3D,E).

Given that macrophages polarize into the M1 phenotype, which provokes inflammation, or into the M2 phenotype, which attenuates inflammation in different immune microenvironments, the changes in the expression of polarization markers were then assessed in light of their known pivotal involvement in hepatic IRI. Compared to that in the control group, the expression of CD206, an M2 polarization marker, exhibited a significant decreasing trend after H/R intervention. However, the level of INOS, an M1 polarization marker, increased following H/R (Figure 3F). Further blocking of PPM1G with lentivirus increased the inclination of markers of macrophage polarization to the M1 phenotype. These in vitro findings suggest that PPM1G may restrict macrophage polarization toward the M1 phenotype by inhibiting the STING pathway and thus alleviating hepatic IRI.

### 3.4 Knockdown of PPM1G aggravated murine hepatic IRI in vivo

**Figure 4:**
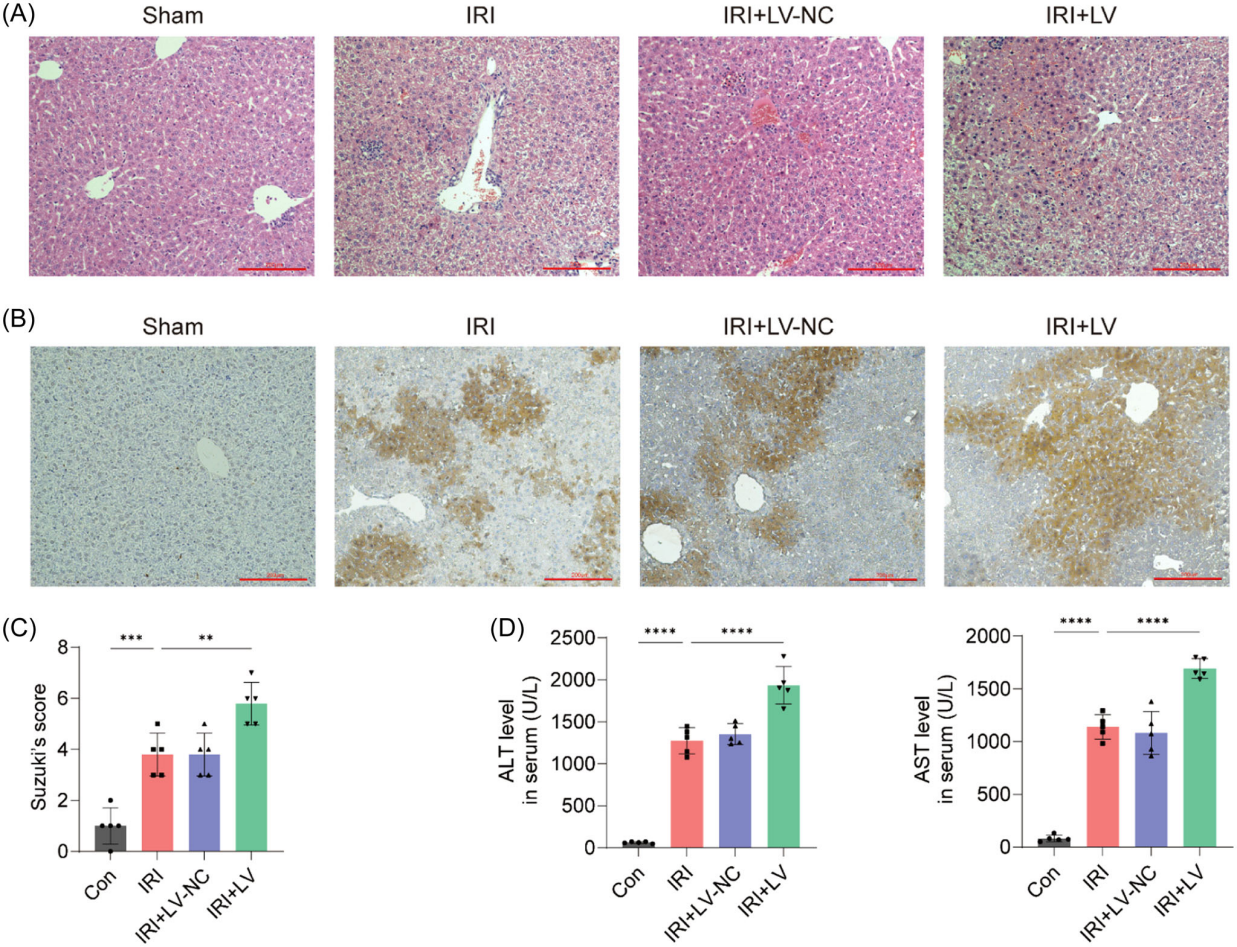
Knockdown of PPM1G attenuated mouse hepatic ischemia/reperfusion injury (IRI) in vivo. (A, C) Hematoxylin and Eosin (H&E) staining and Suzuki's scores of mouse liver tissue. Magnification, ×200; scale bar = 200 μm. (B) TUNEL staining of mouse liver tissue. Magnification, ×200; scale bar = 200 μm. (D) Levels of alanine (ALT) and aspartate transaminase (AST) in mouse serum. Results are expressed as the relative mean and SD ratio of the indicated number of animals (*n* = 5). Statistical significance was assessed by one‐way analysis of variance (ANOVA). **p < .01, ***p < .001 and ****p < .0001, respectively. Con, control; LV, lentivirus; NC, negative control.

To investigate the impact of PPM1G on hepatic IRI in vivo, PPM1G shRNA or NC was injected into the tail vein of mice before 70% warm liver I/R treatment. Consistently, PPM1G deficiency resulted in more severe pathophysiological alterations in liver tissue, as evidenced by H&E staining and Suzuki's score (Figure 4A,C). Similarly, hepatocyte apoptosis increased, as evidenced by TUNEL staining (Figure 4B). Deteriorated liver function was also reflected by elevated serum AST and ALT levels in the IRI + PPM1G shRNA group compared to the group challenged only by IRI (Figure 4D). Taken together, these results suggest that PPM1G is indispensable for limiting I/R‐mediated hepatic injury and that downregulating PPM1G might aggravate IRI in mice.

### 3.5 STING pathway overactivation and M1 macrophage polarization were reversed by C‐176

**Figure 5:**
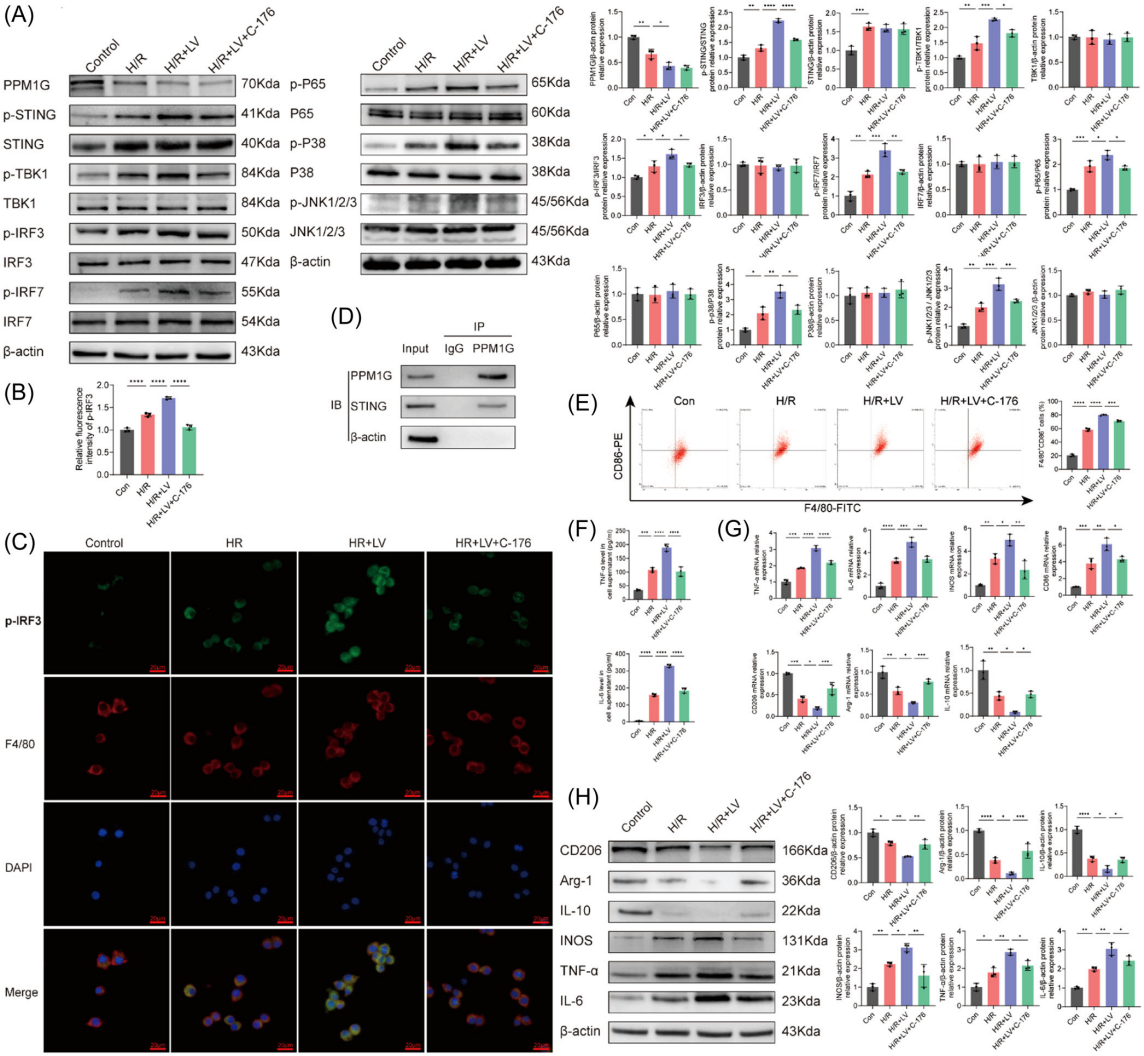
STING pathway overactivation and macrophage M1 polarization were reversed by C‐176. (A) Immunoblotting images and quantitative analysis of PPM1G, p‐STING, STING, p‐TBK1, TBK1, p‐IRF3, IRF3, p‐IRF7, IRF7, p‐P65, P65, p‐P38, P38, p‐JNK1/2/3 and JNK1/2/3 in RAW 264.7 cells. (B, C) Immunofluorescence of p‐IRF3 and F4/80 in RAW 264.7 cells. Magnification, ×200; scale bar = 20 μm. (F) The mRNA expressions of TNF‐α and IL‐6 in RAW 264.7 cells. (D) Protein interaction between PPM1G and STING. (E) Representative flow cytometry plots and statistical analysis of Raw 264.7 cells staining with FITC‐F4/80 and PE‐CD86. (F) Levels of TNF‐α and IL‐6 in cell supernatant. (G) The mRNA expressions of TNF‐α, IL‐6, INOS, CD86, CD206, Arg‐1 and IL‐10 in RAW 264.7 cells. (H) Immunoblotting images and quantitative analysis of macrophage polarization markers and inflammatory factors including CD206, Arg‐1, IL‐10, INOS, TNF‐α, and IL‐6 in RAW 264.7 cells. Results are expressed as the relative mean and SD ratio of three independent sets of experiments. Statistical significance was assessed by one‐way analysis of variance (ANOVA). *p < .05, **p < .01, ***p < .001 and ****p < .0001. Con, control; H, hypoxia; LV, lentivirus; mRNA, messenger RNA; R, reoxygenation.

Having demonstrated that PPM1G modulates functional outcomes after hepatic IRI, C‐176, a classic STING inhibitor that inhibits the palmitoylation of STING at cytoplasmic proximal cysteine residues and prevents the assembly of the STING multimeric complex at the Golgi apparatus in a signaling‐incompetent state,^22^ was applied in vitro to further validate whether PPM1G modulates macrophage polarization through the STING pathway and, eventually, affects hepatic IRI. As shown by immunoblotting, we found that C‐176 administration effectively decreased the protein level of phosphorylated STING, while the total protein level of STING was unaffected. Moreover, the relevant downstream participants of STING, including TBK1, IRF3, and IRF7, exhibited a propensity toward dephosphorylation in the presence of C‐176, manifesting a relief of the promoting effect on inflammatory genes that initiated by STING phosphorylation (Figure 5A). Consistently, immunostaining for phosphorylated IRF3 revealed that the intracellular fluorescence signal intensity was substantially lower in the H/R + LV + C‐176 group than in the H/R + LV group (Figure 5B,C). According to the results of CO‐IP, there is a direct binding between STING and PPM1G proteins, further corroborating the mechanism of regulation of STING phosphorylation modification by PPM1G (Figure 5D). We further checked the possibility that whether PPM1G can modulate STING pathway via modulation of Nrf2‐HO‐1. As shown in Supporting Information S1: Figure S1E, after the intervention of PPM1G‐Lentivirus in hypoxia/regeneration managed RAW 264.7 cells, there was no significant protein expression changes of Nrf2 and HO‐1, basically ruling out the possibility that PPM1G acts through Nrf‐2 or HO‐1 in our model. Notably, phosphorylated p65 also appeared to be affected by STING blockade, further confirming that p65 may participate in cross‐talk with the NF‐κB pathway in hepatic IRI (Figure 5A). The levels of the primary mediators of the mitogen‐activated protein kinase (MAPK) pathway, p38 and Jnk, also showed a downward trend after the administration of c‐176, confirming the involvement of multiple inflammatory pathways downstream of PPM1G‐STING in cross‐talk (Figure 5A). Changes in the levels of TNF‐α and IL‐6 in the cell supernatant were in line with the results of the western blot and qRT‐PCR analyses (Figure 5F–H).

We then investigated the effects of STING repression on macrophage polarization. Restricting STING with C‐176 effectively limited macrophage M1 polarization, as shown by the downregulation of INOS and upregulation of CD206, Arg‐1, and IL‐10 (Figure 5H). Consistently, the mRNA expression of M2 polarization biomarkers, including Arg1, CD206, and IL‐10, tended to increase after treatment with c‐176 (Figure 5G). Moreover, the expression of CD86, another M1 polarization marker, increased under the influence of hypoxia/reoxygenation and further increased significantly after treatment with PPM1G‐specific shRNA, while the addition of C‐176 inhibited the expression of CD86 (Figure 5E). Taken together, these results demonstrated that blockade of STING had the opposite impact on enhanced hepatic IRI driven by PPM1G downregulation through modulating polarization in vitro.

### 3.6 STING blockade improved the deterioration of liver function caused by PPM1G knockdown in vivo

**Figure 6:**
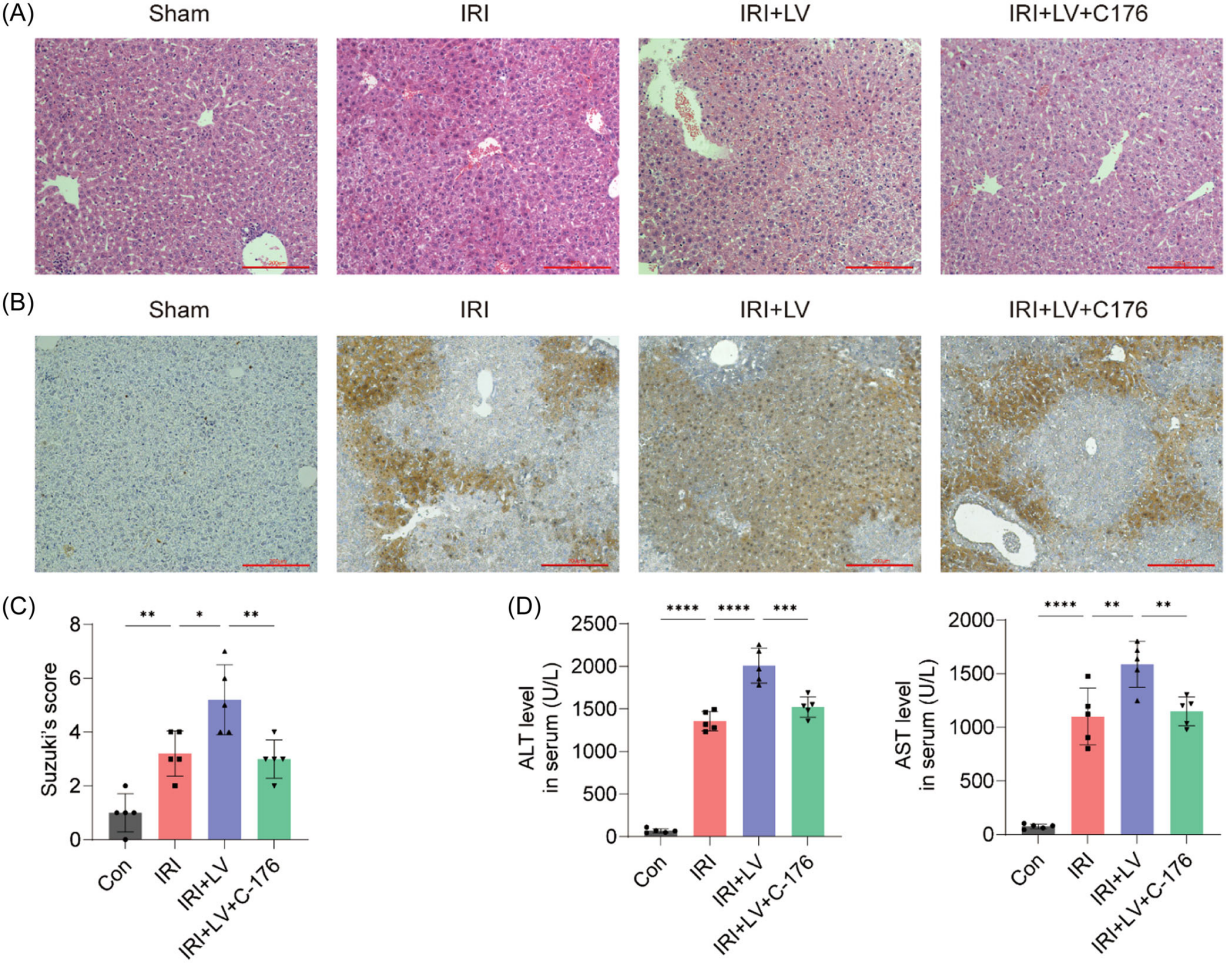
STING blockade improved deteriorated liver function caused by PPM1G knockdown in vivo. (A, C) H&E staining and Suzuki's scores of mouse liver tissue. Magnification, ×200; scale bar = 200 μm. (B) TUNEL staining of mouse liver tissue. Magnification, ×200; scale bar = 200 μm. (D) Levels of alanine transaminase (ALT) and aspartate transaminase (AST) in mouse serum. Results are expressed as the relative mean and SD ratio of the indicated number of animals (*n* = 5). Statistical significance was assessed by one‐way analysis of variance (ANOVA). *p < .05, **p < .01, ***p < .001 and ****p < .0001. Con, control; IRI, ischemia/reperfusion injury; LV, lentivirus.

We further utilized C‐176 in vivo to assess the changes in liver pathology caused by STING blockade in mice with hepatic IRI. We found that the hepatic pathological damage and apoptosis elicited by PPM1G repression were rescued by intraperitoneal C‐176 preadministration (Figure 6A–C). In addition, the introduction of c‐176 drastically reversed the stimulatory effects of PPM1G knockdown on the serum AST and ALT concentrations (Figure 6D), demonstrating the recovery of physiological liver function.

## 4 DISCUSSION

**Figure 7:**
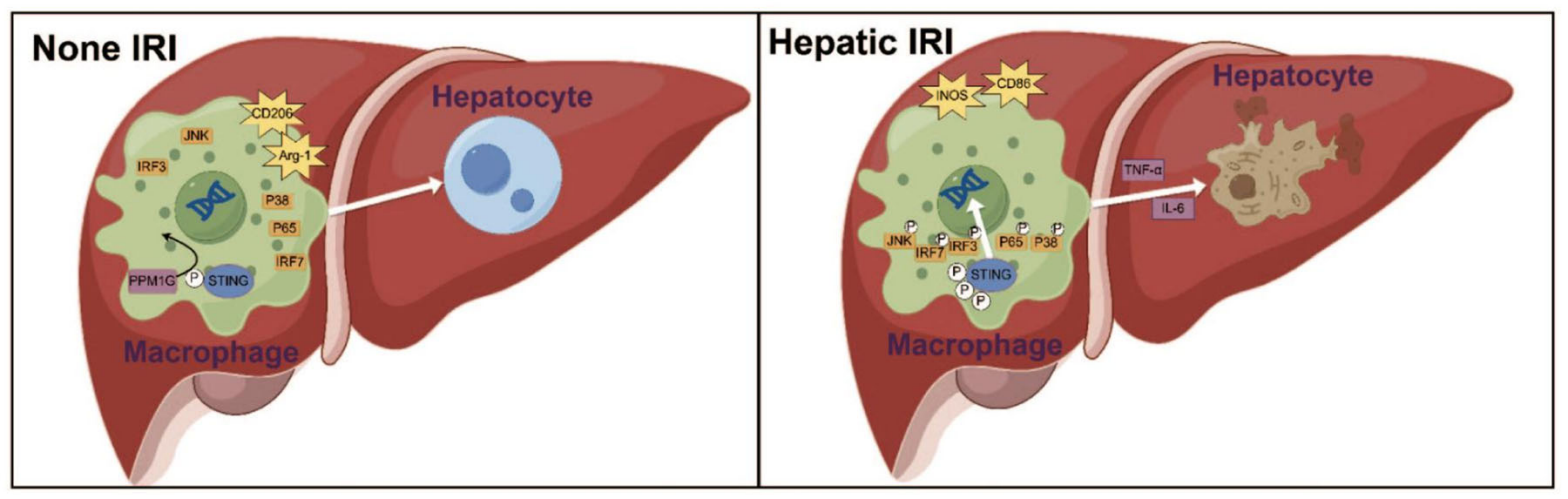
Schematic illustration of hepatic ischemia/reperfusion injury (IRI) downregulating PPM1G, inducing STING phosphorylation and eventually promoting KC M1 polarization.

Due to the necessity of a series of surgical maneuvers, such as portal vein blockade and revascularization, IRI is inevitable for almost all liver surgeries, including liver transplantation. Although a number of hypothesized mechanisms for hepatic IRI have emerged in recent years, dysregulated nonparenchymal cells, especially overactivated macrophages, inside the hepatic circulation have been recognized to play a crucial role in mediating hepatic IRI. Moreover, although few members of the PPM family of proteins have been implicated in liver cirrhosis and hepatocellular carcinoma, the role of PPM1G in hepatic IRI has not been determined. Therefore, in the present work, we identified PPM1G as a potent regulator of hepatic IRI by restricting KC M1 polarization by maintaining STING activation balanced by dephosphorylation (Figure 7).

STING is an endoplasmic reticulum (ER)‐located transmembrane protein downstream of cyclic GMP–AMP synthase (cGAS), a cytoplasmic sensor that is activated to synthesize the particular second messenger cyclic GMP–AMP (cGAMP) from ATP or GTP released from ribosomes or from nuclear lysis.^26^, ^27^ STING dissociates from the ER after binding to cGAMP and proceeds to the Golgi apparatus, where TBK1 is recruited and able to autophosphorylate itself. Thereafter, phosphorylated TBK1, in turn, enriches STING to form oligomers that autophosphorylate themselves at a conserved motif that serves as a docking site for IRF3 phosphorylation.^28^ Dimerized p‐IRF3 eventually penetrates the nucleus, where type I IFN production is stimulated. Moreover, although it can be directly activated by Toll‐like receptor 9, IRF7 strongly connects with the STING pathway.^25^, ^29^ The STING pathway was initially shown to contribute to innate immunity against exogenous pathogens. Nevertheless, many recent studies have shown that STING also plays an indelible role in diverse inflammatory responses. As previously reported, STING has a relationship with upstream noncoding miRNAs, which base pair with downstream STING mRNA, culminating in reduced type I IFN production and restoration of liver function.^30^ Moreover, calcium‐dependent caspase 1‐GSDMD‐mediated pyroptosis and the NLRP3‐mediated inflammasome were amplified following STING activation in hepatic IRI.^31^, ^32^ However, not exclusive to the liver, the STING pathway appears to be overactivated as an accelerator of IRI in multiple other organs, including the brain and gut.^33^, ^34^ Therefore, a comprehensive investigation of putative mechanisms that precisely regulate the STING pathway is needed to ensure proper and balanced innate immune homeostasis.

In addition, a recent study revealed that PPM1G represses STING by dephosphorylating,^24^ which led us to hypothesize that PPM1G may also restrict hepatic IRI in a typical manner. A classic STING suppressant, c‐176, was therefore introduced for further in‐depth exploration of the connection between PPM1G and STING. Indeed, the changes in the protein expression of p‐TBK1, p‐IRF3, and p‐IRF7 were almost abrogated after C‐176 administration, as shown by the western blot results. STING inhibition with C‐176 attenuated the secretion of pro‐inflammatory cytokines by BMDMs in aged mice.^32^ In parallel with these results, decreases in the concentrations of TNF‐α and IL‐6 were detected by ELISA, qRT‒PCR and western blot analysis in the H/R + PPM1G‐LV group after pharmacological inhibition of STING. STING can activate multiple downstream inflammatory pathways, including MAPK and NF‐κB pathways; however, p65, the master transcription factor of NF‐κB, maintains a sophisticated interaction with the PPM1G–STING axis, so the overall effect is enigmatic. PPM1G is an NF‐κB coactivator that dephosphorylates CDK9 and subsequently releases 7SK‐unbound P‐TEFb, subsequently facilitating the transcription of NF‐κB target genes.^24^, ^35^ Moreover, p65 phosphorylation is usually followed by p‐IRF3 accumulation.^36^, ^37^, ^38^ Intriguingly, we observed that the expression of p‐p65 increased under H/R conditions and thereafter decreased following c‐176 treatment. This might be because, on the one hand, PPM1G functions primarily as a phosphorylase in the cytoplasm, obscuring its minor role in the nucleus. On the other hand, the cumulative effect of STING overactivation on p65 expression in hepatic IRI outweighs the effect of PPM1G, resulting in an increase in p‐p65. Considering that the NF‐κB pathway is composed of several transcription factors with critical effects, the exact mechanism by which IRF‐3 affects p65 remains unexplained, compelling us to investigate this topic in the future.

Despite the observation that an irritant of IRI was capable of impeding M2 polarization in light of M2 markers, confirming prior findings,^9^, ^39^ another significant finding from our work was that PPM1G could restrain M1 polarization by inhibiting STING. The STING pathway has been shown to govern microglial polarization by upregulating both IRF3 and NF‐κB expression after ischemic stroke.^36^, ^40^ Since the same process was anticipated to be feasible for hepatic IRI treatment, western blot analysis and flow cytometry were used to evaluate changes in the expression of polarization markers in RAW264.7 cells in different groups. Eventually, downregulation of PPM1G, coupled with augmented STING phosphorylation, promoted macrophage phenotypic polarization to M1, as expected, whereas injection of c‐176 restricted the M1 phenotype. It is generally understood that imbalanced macrophage polarization contributes to the exacerbation of IRI progression.^41^, ^42^ In the present work, we found that PPM1G not only acts as a balancer of STING activation but also plays a role as a balancer of macrophage polarization, indicating that any method for effectively restoring PPM1G expression might be a promising therapeutic approach for hepatic IRI.

This study has several limitations. First, although RAW 264.7 cells are a recognized alternative cell line for liver macrophages, primary KCs from liver tissues were not used in this work. The outcomes of these experiments may be biased in particular ways because of subtle physiological variations across cell lines. This work could be more convincing if a coculture model of H/R injury was used in which macrophages and liver cells were utilized. Second, this study lacked certain information on ischemic human liver tissue, such as the expression of PPM1G and STING. As a result, the clinical application of these methods in the current work is somewhat limited. Third, without additional examination of the impact of this modification on other inflammatory pathways affected by phosphorylation, the research effort was limited to the effect of PPM1G on the phosphorylation of the STING pathway in the setting of hepatic IRI. We will focus on this topic in future work.

## 5 CONCLUSION

Our findings indicated that PPM1G regulates hepatic IRI and macrophage polarization through the STING‐mediated inflammatory pathways, revealing that PPM1G is a potential biomarker and target for therapy.

## AUTHOR CONTRIBUTIONS

**Dadi Peng**: Conceptualization; data curation; formal analysis; investigation; writing—original draft. **Zuotian Huang**: Methodology; validation; visualization. **Hang Yang**: Resources; software. **Yunhai Luo**: Data curation; validation. **Zhongjun Wu**: Supervision.

## Supporting information

Supporting information.

## Data Availability

The datasets used and analyzed during the current study are available from the corresponding author upon reasonable request.

## References

[1] Sapisochin G , Bruix J . Liver transplantation for hepatocellular carcinoma: outcomes and novel surgical approaches. Nat Rev Gastroenterol Hepatol. 2017;14(4):203‐217. 10.1038/nrgastro.2016.193 28053342

[2] Masior Ł , Grąt M . Methods of attenuating ischemia‐reperfusion injury in liver transplantation for hepatocellular carcinoma. Int J Mol Sci. 2021;22(15):8229. 10.3390/ijms22158229 34360995 PMC8347959

[3] Bi X , Du C , Wang X , et al. Mitochondrial damage‐induced innate immune activation in vascular smooth muscle cells promotes chronic kidney disease‐associated plaque vulnerability. Adv Sci. 2021;8(5):2002738. 10.1002/advs.202002738 PMC7927614 33717842

[4] Ye L , He S , Mao X , Zhang Y , Cai Y , Li S . Effect of hepatic macrophage polarization and apoptosis on liver ischemia and reperfusion injury during liver transplantation. Front Immunol. 2020;11:1193. 10.3389/fimmu.2020.01193 32676077 PMC7333353

[5] Hu Y , Yang C , Shen G , et al. Hyperglycemia‐triggered sphingosine‐1‐phosphate and sphingosine‐1‐phosphate receptor 3 signaling worsens liver ischemia/reperfusion injury by regulating M1/M2 polarization. Liver Transpl. 2019;25(7):1074‐1090. 10.1002/lt.25470 30972941 PMC6617772

[6] Krenkel O , Tacke F . Liver macrophages in tissue homeostasis and disease. Nat Rev Immunol. 2017;17(5):306‐321. 10.1038/nri.2017.11 28317925

[7] Dey A , Allen J , Hankey‐Giblin PA . Ontogeny and polarization of macrophages in inflammation: blood monocytes versus tissue macrophages. Front Immunol. 2015;5:683. 10.3389/fimmu.2014.00683 25657646 PMC4303141

[8] Wang H , Xi Z , Deng L , Pan Y , He K , Xia Q . Macrophage polarization and liver ischemia‐reperfusion injury. Int J Med Sci. 2021;18(5):1104‐1113. 10.7150/ijms.52691 33526969 PMC7847630

[9] Huang Z , Mou T , Luo Y , et al. Inhibition of miR‐450b‐5p ameliorates hepatic ischemia/reperfusion injury via targeting CRYAB. Cell Death Dis. 2020;11(6):455. 10.1038/s41419-020-2648-0 32532961 PMC7293338

[10] Rao J , Cheng F , Zhou H , et al. Nogo‐B is a key mediator of hepatic ischemia and reperfusion injury. Redox Biol. 2020;37:101745. 10.1016/j.redox.2020.101745 33099216 PMC7582106

[11] Gudipaty SA , McNamara RP , Morton EL , D'Orso I . PPM1G binds 7SK RNA and Hexim1 to block P‐TEFb assembly into the 7SK snRNP and sustain transcription elongation. Mol Cell Biol. 2015;35(22):3810‐3828. 10.1128/MCB.00226-15 26324325 PMC4609742

[12] Petri S , Grimmler M , Over S , Fischer U , Gruss OJ . Dephosphorylation of survival motor neurons (SMN) by PPM1G/PP2Cγ governs cajal body localization and stability of the SMN complex. J Cell Biol. 2007;179(3):451‐465. 10.1083/jcb.200704163 17984321 PMC2064792

[13] Ryu EJ , Angelastro JM , Greene LA . Analysis of gene expression changes in a cellular model of parkinson disease. Neurobiol Dis. 2005;18(1):54‐74. 10.1016/j.nbd.2004.08.016 15649696

[14] Liu J , Stevens PD , Eshleman NE , Gao T . Protein phosphatase PPM1G regulates protein translation and cell growth by dephosphorylating 4E binding protein 1 (4E‐BP1). J Biol Chem. 2013;288(32):23225‐23233. 10.1074/jbc.M113.492371 23814053 PMC3743494

[15] Xu K , Wang L , Feng W , Feng Y , Shu HK . Phosphatidylinositol‐3 kinase‐dependent translational regulation of Id1 involves the PPM1G phosphatase. Oncogene. 2016;35(44):5807‐5816. 10.1038/onc.2016.115 27065332 PMC5064830

[16] Pyo J , Ryu J , Kim W , Choi JS , Jeong JW , Kim JE . The protein phosphatase PPM1G destabilizes HIF‐1α expression. Int J Mol Sci. 2018;19(8):2297. 10.3390/ijms19082297 30081604 PMC6121667

[17] Ge M , Liu H , Zhang N , et al. Costunolide represses hepatic fibrosis through WW domain‐containing protein 2‐mediated Notch3 degradation. Br J Pharmacol. 2020;177(2):372‐387. 10.1111/bph.14873 31621893 PMC6989949

[18] Chen D , Zhao Z , Chen L , Li Q , Zou J , Liu S . PPM1G promotes the progression of hepatocellular carcinoma via phosphorylation regulation of alternative splicing protein SRSF3. Cell Death Dis. 2021;12(8):722. 10.1038/s41419-021-04013-y 34290239 PMC8295330

[19] Lin YR , Yang WJ , Yang GW . Prognostic and immunological potential of PPM1G in hepatocellular carcinoma. Aging. 2021;13(9):12929‐12954. 10.18632/aging.202964 33952716 PMC8148464

[20] Yang M , Zhang M , Nakajima H , Yudasaka M , Iijima S , Okazaki T . Time‐dependent degradation of carbon nanotubes correlates with decreased reactive oxygen species generation in macrophages. Int J Nanomedicine. 2019;14:2797‐2807. 10.2147/IJN.S199187 31118611 PMC6501421

[21] Pardo V , González‐Rodríguez Á , Guijas C , Balsinde J , Valverde ÁM . Opposite cross‐talk by oleate and palmitate on insulin signaling in hepatocytes through macrophage activation. J Biol Chem. 2015;290(18):11663‐11677. 10.1074/jbc.M115.649483 25792746 PMC4416868

[22] Haag SM , Gulen MF , Reymond L , et al. Targeting STING with covalent small‐molecule inhibitors. Nature. 2018;559(7713):269‐273. 10.1038/s41586-018-0287-8 29973723

[23] Liu Y , Lu T , Zhang C , et al. Activation of YAP attenuates hepatic damage and fibrosis in liver ischemia‐reperfusion injury. J Hepatol. 2019;71(4):719‐730. 10.1016/j.jhep.2019.05.029 31201834 PMC6773499

[24] Yu K , Tian H , Deng H . PPM1G restricts innate immune signaling mediated by STING and MAVS and is hijacked by KSHV for immune evasion. Sci Adv. 2020;6(47):eabd0276. 10.1126/sciadv.abd0276 33219031 PMC7679160

[25] Negishi H , Taniguchi T , Yanai H . The interferon (IFN) class of cytokines and the IFN regulatory factor (IRF) transcription factor family. Cold Spring Harbor Perspect Biol. 2018;10(11):a028423. 10.1101/cshperspect.a028423 PMC6211389 28963109

[26] Wu J , Sun L , Chen X , et al. Cyclic GMP‐AMP is an endogenous second messenger in innate immune signaling by cytosolic DNA. Science. 2013;339(6121):826‐830. 10.1126/science.1229963 23258412 PMC3855410

[27] Hopfner KP , Hornung V . Molecular mechanisms and cellular functions of cGAS‐STING signalling. Nat Rev Mol Cell Biol. 2020;21(9):501‐521. 10.1038/s41580-020-0244-x 32424334

[28] Zhang C , Shang G , Gui X , Zhang X , Bai X , Chen ZJ . Structural basis of STING binding with and phosphorylation by TBK1. Nature. 2019;567(7748):394‐398. 10.1038/s41586-019-1000-2 30842653 PMC6862768

[29] Suschak JJ , Wang S , Fitzgerald KA , Lu S . A cGAS‐independent STING/IRF7 pathway mediates the immunogenicity of DNA vaccines. J Immunol. 2016;196(1):310‐316. 10.4049/jimmunol.1501836 26590319 PMC4685033

[30] Shen A , Zheng D , Luo Y , et al. MicroRNA‐24‐3p alleviates hepatic ischemia and reperfusion injury in mice through the repression of STING signaling. Biochem Biophys Res Commun. 2020;522(1):47‐52. 10.1016/j.bbrc.2019.10.182 31735332

[31] Wu X , Chen Y , Liu C , Gong J , Xu X . STING induces liver ischemia‐reperfusion injury by promoting calcium‐dependent caspase 1‐GSDMD processing in macrophages. Oxid Med Cell Longevity. 2022;2022:1‐19. 10.1155/2022/8123157 PMC8906939 35281468

[32] Zhong W , Rao Z , Rao J , et al. Aging aggravated liver ischemia and reperfusion injury by promoting STING‐mediated NLRP3 activation in macrophages. Aging cell. 2020;19(8):e13186. 10.1111/acel.13186 32666684 PMC7431827

[33] Liao Y , Cheng J , Kong X , et al. HDAC3 inhibition ameliorates ischemia/reperfusion‐induced brain injury by regulating the microglial cGAS‐STING pathway. Theranostics. 2020;10(21):9644‐9662. 10.7150/thno.47651 32863951 PMC7449914

[34] Wu J , Liu Q , Zhang X , Wu X , Zhao Y , Ren J . STING‐dependent induction of lipid peroxidation mediates intestinal ischemia‐reperfusion injury. Free Radic Biol Med. 2021;163:135‐140. 10.1016/j.freeradbiomed.2020.12.010 33347986

[35] McNamara RP , McCann JL , Gudipaty SA , D'Orso I . Transcription factors mediate the enzymatic disassembly of promoter‐bound 7SK snRNP to locally recruit P‐TEFb for transcription elongation. Cell Rep. 2013;5(5):1256‐1268. 10.1016/j.celrep.2013.11.003 24316072 PMC3882317

[36] Kong L , Li W , Chang E , et al. mtDNA‐STING axis mediates microglial polarization via IRF3/NF‐κB signaling after ischemic stroke. Front Immunol. 2022;13:860977. 10.3389/fimmu.2022.860977 35450066 PMC9017276

[37] Luo X , Li H , Ma L , et al. Expression of STING is increased in liver tissues from patients with NAFLD and promotes macrophage‐mediated hepatic inflammation and fibrosis in mice. Gastroenterology. 2018;155(6):1971‐1984. 10.1053/j.gastro.2018.09.010 30213555 PMC6279491

[38] Cai H , Yan L , Liu N , Xu M , Cai H . IFI16 promotes cervical cancer progression by upregulating PD‐L1 in immunomicroenvironment through STING‐TBK1‐NF‐kB pathway [published correction appears in Biomed Pharmacother]. Biomed Pharmacother. 2020;123:109790. 10.1016/j.biopha.2019.109790 31896065

[39] Chu X , Cao L , Yu Z , et al. Hydrogen‐rich saline promotes microglia M2 polarization and complement‐mediated synapse loss to restore behavioral deficits following hypoxia‐ischemic in neonatal mice via AMPK activation. J Neuroinflamm. 2019;16(1):104. 10.1186/s12974-019-1488-2 PMC6525972 31103039

[40] Zhao S , Ma L , Chu Z , Xu H , Wu W , Liu F . Regulation of microglial activation in stroke. Acta Pharmacol Sin. 2017;38(4):445‐458. 10.1038/aps.2016.162 28260801 PMC5386316

[41] Zhang M , Nakamura K , Kageyama S , et al. Myeloid HO‐1 modulates macrophage polarization and protects against ischemia‐reperfusion injury. JCI Insight. 2018;3(19):e120596. 10.1172/jci.insight.120596 30282830 PMC6237471

[42] Liu Y , Zhang W , Cheng Y , Miao C , Gong J , Wang M . Activation of PPARγ by curcumin protects mice from ischemia/reperfusion injury induced by orthotopic liver transplantation via modulating polarization of Kupffer cells. Int Immunopharmacol. 2018;62:270‐276. 10.1016/j.intimp.2018.07.013 30036770

