# Supplementary material for: PPM1G regulates hepatic ischemia/reperfusion injury through STING‐mediated inflammatory pathways in macrophages
Source: Immun Inflamm Dis. 2024 Feb 19;12(2):e1189. doi: 10.1002/iid3.1189 (PMC10875902; doi:10.1002/iid3.1189)
Supplement: Supplementary file 1 — Supporting information. [file IID3-12-e1189-s001.docx]

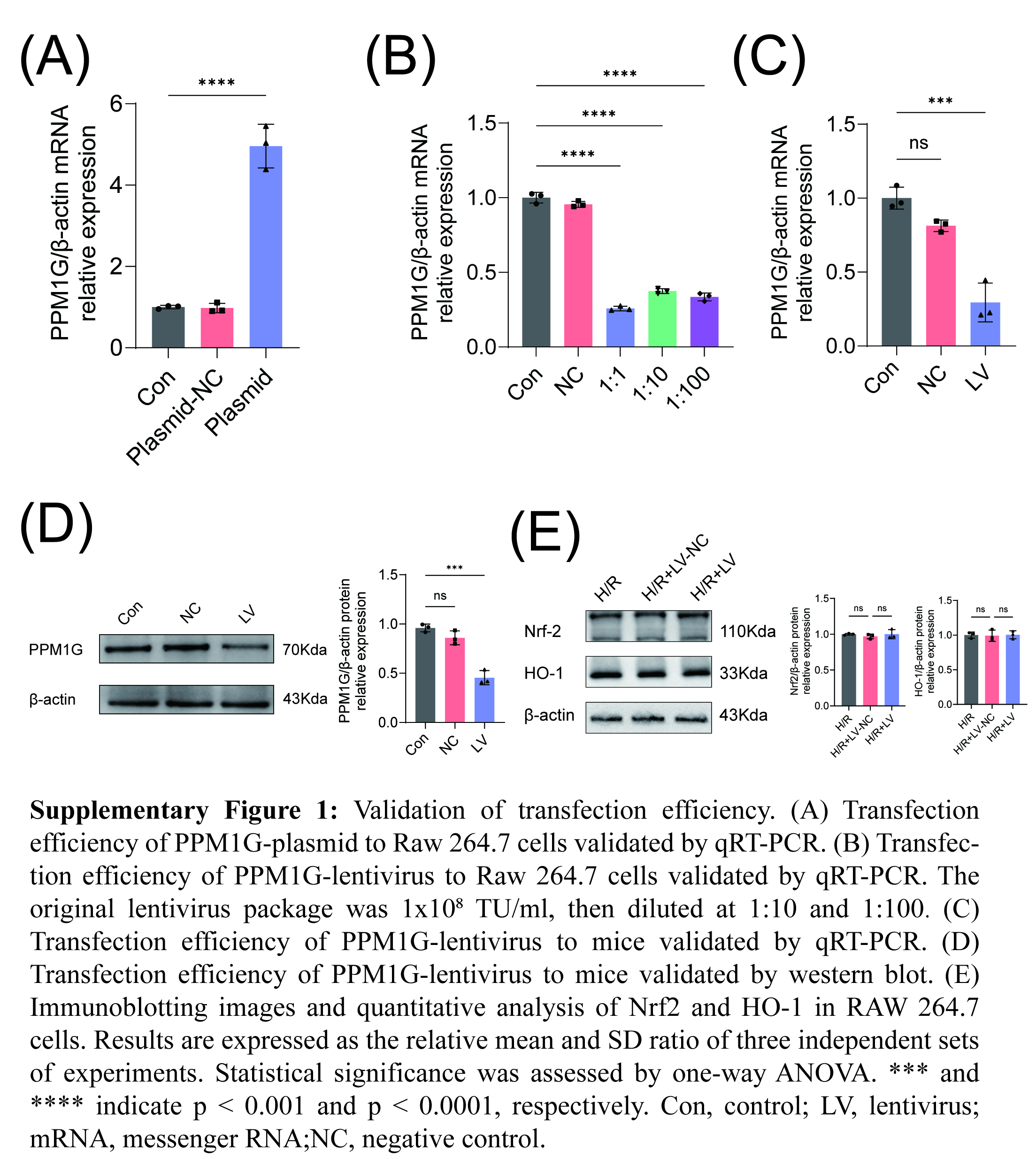


| **Supplementary Table 1.** Antibody dilutions for western blot. |  |
| --- | --- |
| Antibody name | Dilution |
| PPM1G, STING, TBK1, IRF3, IRF7, P65, TNF-α, IL-6, P38, JNK1/2/3, HO-1, Arg-1, β-actin | 1:1000 |
| p-STING, p-TBK1, p-IRF3, p-IRF7, INOS, CD206, p-P65, p-P38, p-JNK1/2/3, IL-10, Nrf2 | 1:500 |
